# Long-Term Effect of Environmental Enrichment on Reproductive Performance of Swiss Webster Mice and Their Female Offspring

**DOI:** 10.3390/ani10081438

**Published:** 2020-08-18

**Authors:** María Noel Meikle, Ana Paula Arévalo, Geraldine Schlapp, Gabriel Fernández-Graña, Alejo Menchaca, Martina Crispo

**Affiliations:** 1 Unidad de Animales Transgénicos y de Experimentación (UATE), Institut Pasteur de Montevideo, Mataojo 2020, Montevideo, CP 11400， Uruguay; manomeikle@pasteur.edu.uy (M.N.M.); aparevalo@pasteur.edu.uy (A.P.A.); gschlapp@pasteur.edu.uy (G.S.); gabifergra@pasteur.edu.uy (G.F.-G.); 2 Instituto de Reproducción Animal Uruguay, Cno Cruz del Sur 2250 Montevideo 12200, Uruguay; menchaca.alejo@gmail.com

**Keywords:** animal welfare, 3Rs, animal facilities, reproduction, murine

## Abstract

**Simple Summary:**

The aim of this study was to evaluate the effect of the implementation of an environmental enrichment (EE) plan on reproductive performance in mice, applied during one year period in Swiss Webster breeders and their female offspring used as recipients for embryo transfer. Birth rate, litter size, pups’ weight at weaning, interlitter interval and time to first litter were compared against a control group that did not receive EE. In mice breeders, EE significantly increased pups’ weight compared to control group (*p* < 0.01), without changing any other reproductive parameters. In the offspring females used as recipients, EE did not modify reproductive parameters. Our results demonstrate that this enrichment plan, which is usually applied in mouse facilities for animal well-being, enhances pup’s weight at weaning, with no negative effects on the reproductive performance of Swiss Webster mice.

**Abstract:**

The aim of this study was to evaluate the effect of an environmental enrichment (EE) plan on the reproductive performance of Swiss Webster mice and their female offspring used as recipients for embryo transfer. A total of 54 breeder mice and 60 F1 females, used as foster mothers, were allocated in two experimental groups to receive or not receive EE for physical well-being. Reproductive outcomes of the Swiss trios such as birth rate and pup number, litter size, pups’ weight at weaning, interlitter interval and time to first litter were analyzed. Environmental enrichment significantly increased pups weight from breeding trios compared to the control group (14.4 ± 0.1 vs. 13.8g ± 0.1, EE vs. control, respectively; *p* < 0.01). Other parameters did not differ between both groups. Reproductive parameters of female offspring used as recipients for embryo transfer did not differ among groups subjected or not to EE. These data demonstrate that the EE protocol applied in Swiss Webster breeder mice positively enhanced pups weight, and did not interfere with other reproductive outcomes. In conclusion, this study supports the implementation of EE plans usually applied for animal welfare in mouse facilities with slight improvement in reproductive performance.

## 1. Introduction

Animal welfare encompasses society’s expectations regarding the conditions that animals should experience when under human control. According to international frameworks, captive, experimental and domestic animals should maintain their freedom to express normal patterns of behavior [1,2]. Environmental enrichment (EE) is defined as any modification in the environment of captive animals that seeks to enhance its physical and psychological well-being by providing stimuli that meets the animal’s species-specific needs to express its behavior [3]. Even though laboratory rodents have been adapted to confined life, their environment should contemplate innate physiological and behavioral needs typical of the species, such as their wild counterparts. For that reason, social contacts, gnawing, resting, hiding, exploring, foraging and nest building should be available in their environment.

Environmental conditions have a significant impact on the well-being of laboratory animals allowing them to manage the ability to cope with challenges [4]. The implementation of EE promotes the refinement of the 3Rs principle [5,6] and thus, it is a desirable strategy to be used in animal facilities. Environmental enrichment can influence several physiological, neurochemical and behavioral parameters of the animals. Some reports have shown a protective effect of EE in the stress response and anxiety related behaviors [7,8,9]. Normal reproductive functions require expressing natural behavior and absence of stress [10], so it is interesting to investigate the effect of EE on reproduction. Moreover, appropriate reproductive performance is metabolically costly and requires favorable energy balance and stress-free condition, thus, reproductive success is a good indicator of well-being in several species [11].

Although some studies have revealed a positive correlation between EE and reproduction in mice [12,13,14], the information is scare, controversial and requires further investigation. Previous reports have shown better outcomes in the number of pups weaned per dam and pups weaning weight in three different mice strains when nesting materials were provided, compared to the control group [15]. Additionally, it has been found that EE promotes a greater number of viable oocytes induced by superovulation in rats [16]. On the other hand, other authors did not find any effect of EE on reproductive parameters [17,18]. Despite these findings, further information is required on the effect of EE on reproduction in mice breeding colonies and their progeny.

In order to provide further information on the effect of EE on mice reproductive performance, the aim of this study was to evaluate the long-term effect of an EE plan in Swiss Webster breeder mice and its female offspring used as recipients for embryo transfer. We evaluate reproductive outcomes in terms of number of births and pups, litter size, pups’ weight at weaning, time to first litter and interlitter interval.

## 2. Materials and Methods

### 2.1. Animals

The study involved a total of 114 Swiss Webster stock mice (breeded in-house), housed under specific pathogen-free conditions in individually ventilated cages (IVC, 365 × 207 × 130 mm; Tecniplast, Milan, Italy) at the Transgenic and Experimental Animal Unit of Institut Pasteur de Montevideo. The animals received free access to autoclaved food (Labdiet 5K67, St Louis, MO, USA) and filtered and autoclaved fresh water. Environmental conditions were controlled as follow: temperature ranged between 20–22 °C, relative humidity ranged between 45–65% and photoperiod was 14:10 h light:dark ratio. The experimental protocol was approved by the Institutional Animal Ethics Committee, in accordance with National Law 18.611 and international Guide for the Care and Use of Laboratory Animals [1], regarding laboratory animal’s protocols.

### 2.2. Experimental Design

**Figure 1:**
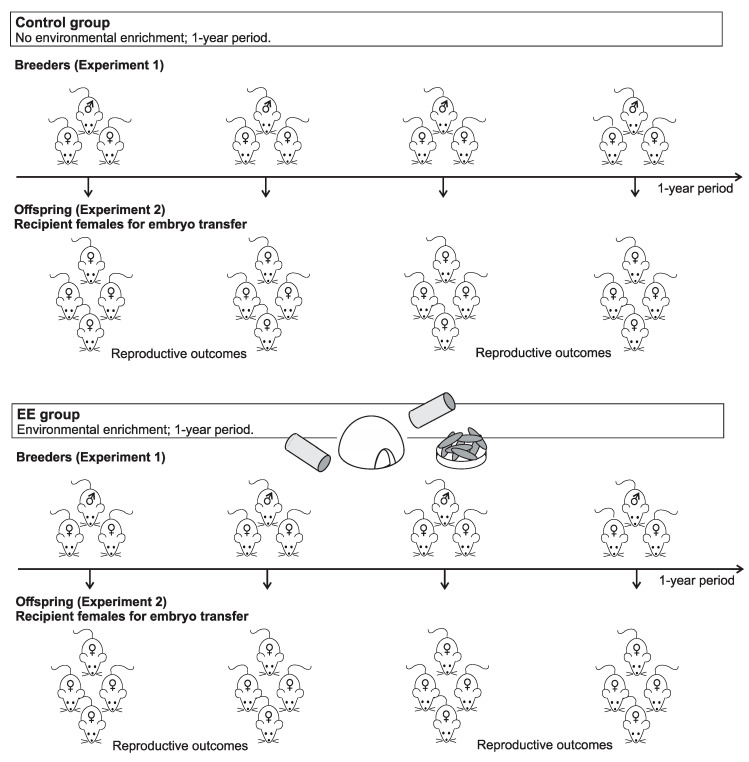
Schematic representation of the experimental design. In Experiment 1, Swiss Webster breeder trios were exposed (environmental enrichment (EE) group) or not (control group) to an environmental enrichment (EE) program. During 1-year period a total of 18 trios (nine per experimental group) were compared throughout their reproductive life. Reproductive outcomes such as time to first litter, interlitter interval, birth rate, litter size, number of pups weaned and pup’s weight were registered. In Experiment 2, female offspring that were born from Experiment 1 (i.e., with or without EE) were used as recipients for embryo transfer. Recipient females continued with (EE group, *n* = 35) or without (control group, *n* = 25) EE during growth, embryo transfer, pregnancy and delivery until pup’s weaning. Pregnancy and birth rate, litter size number and number of pups weaned were compared between both experimental groups.

Two experiments were carried out in breeding mice (Experiment 1) and female recipients (Experiment 2). Experiment 1 was conducted during a one-year period, and Experiment 2 was conducted in the female offspring derived from Experiment 1. In both experiments, the animals were subjected to enrichment conditions or maintained as control group (EE and control group, respectively). Animals were randomly allocated in each experimental group and data were registered by the same animal caretaker during the whole period, in an unblinded manner. A schematic representation of the experimental design is depicted in Figure 1.

### 2.3. Enrichment Protocol

For both experimental groups (control and EE groups), individually ventilated cages were provided with 200 gr wood shavings (local supplier) and two paper towels (2.6 g, Elite, IPUSA S.A., UY), as usually used in standard operative procedures in our mice facility.

For EE groups, cages were provided during the weekly routine husbandry with one or two of the following items combined and changed weekly: 1/2 unit of wholesome treats (BioServ, Flemington, NJ, USA), six units of certified sunflower seeds (local supplier), ten units of certified freeze-dried mealworms (BioServ, Flemington, NJ, USA), four units of fruit bites (BioServ, Flemington, NJ, USA), ten units of certified sucrose reward tablets apple-chocolate flavor (TestDiet, St Louis, MO, USA), one mouse igloo (4 1/4” diameter × 2 1/4” tall) (BioServ, Flemington, NJ, USA), one PVC cylindrical tunnel (2.3” diameter × 3.94” large; local supplier) and one cardboard tube (2” diameter × 3.94” large; local supplier).

### 2.4. Experiment 1: Breeding Mice

A total of 18 animals (8-weeks old, 25 g average), were randomly assigned to conform six trios (i.e., each trio: one male and two females). Three trios were provided with materials for physical enrichment (nesting material and nutritional enhancement), in different combinations during the weekly cage change, as described above (EE group). The remaining three trios received only paper towels weekly (control group). Three replicates were performed throughout the year, changing the trios every four months. Breeder´s cages were regularly checked for pregnancy and delivery. Pups from new litters were registered and the date and number of births recorded. On day 21, pups were weaned, separated by gender and the weight of each animal was measured. Litter number and interlitter interval was recorded for each dam. Female offspring continued or not with EE after weaning, to be used in Experiment 2.

### 2.5. Experiment 2: Female Offspring Used as Embryo Recipients

The objective was to study whether EE applied on the female offspring (used as recipients for embryo transfer) derived from mice that had also received EE has any effect on reproductive performance. A total of 35 and 25 recipient females (8-weeks old, 25 g average), with or without EE, derived from experiment 1, received 846 and 575 embryos, respectively. Embryos were obtained from C57BL/6J or B6D2F1 females treated under standard procedures without EE, and were randomly allocated into experimental groups. Enrichment/non-enrichment protocol continued after embryo transfer, until the weaning of pups resulting from transferred embryos. Pregnancy rate (pregnant/transferred females), litter size number, number of pups weaned, pups gender ratio and birth rate (pups born/transferred embryos) were analyzed for each group.

### 2.6. Embryo Transfer in Recipient Females

Female mice weaned from mothers subjected to enrichment or non-enrichment conditions from Experiment 1 were housed together in groups of six until embryo transfer (8 weeks-old). Before embryo transfer, the recipients were mated with a vasectomized male and positive plug females were transferred with different stage embryos (e.g., either zygotes or morulae). ET was conducted in a randomly blocked design for each treatment (EE and control group), regarding embryo stage (956 zygotes and 465 morulae) and the operator (two trained technicians). Embryo transfer, surgical care and pain relief with 1 mg/kg Tolfenamic acid was performed as previously described [19,20]. Embryos were loaded into a pulled glass pipette and placed through the infundibulum into both oviducts, delivering in average 25–30 zygotes or 17–20 morulae per female.

Afterwards, females were housed in pairs and their cages received the same enrichment/non-enrichment protocol as their original breeding colony during the weekly routine husbandry, until the pups were weaned. If the female number was odd, the remaining female was placed with another conspecific from the same experimental group (without surgery). Pregnancy diagnosis was determined by visual inspection by an experienced animal caretaker two weeks after embryo transfer and litter size was recorded 21 days after birth.

### 2.7. Statistical Analysis

Data collected from three replicates performed in the breeding colony and from the study conducted in foster females were analyzed by logistic regression in Generalized Linear Mixed Models of InfoStat [21]. Data were checked for normality and homogeneity of variance by histograms, q-q plots and formal statistical tests as part of the univariate procedure. The type of variance-covariance structures was chosen depending on the magnitude of the Akaike information criterion (AIC) for models run under heterogeneous compound symmetry, unstructured, autoregressive, spatial power, and first-order ante-dependence. The model with the lowest AIC was chosen. Data were analyzed using the enrichment/non enrichment conditions as fixed factor, as well as replicate, embryo stage (zygotes or morulae) and operator were included in the model. Replicate, embryo stage and operator had no effect on the results (*p* = NS), and the data shown are pooled for these factors. Differences were considered to be significant when *p* < 0.05 and *p* < 0.1 was assumed as a tendency.

## 3. Results

### 3.1. Experiment 1: Breeding Colony

Enrichment conditions in breeding colony neither affected the production of litter pups nor the total number of parturitions per female (Table 1). Additionally, time to first litter (average 22.8 vs. 24.4 days, enrichment vs. control) and interlitter interval (32.7 vs. 36.5 days, enrichment/control) was not different for both treatments (*p* = NS). Similarly, total offspring number as well as the sex number did not differ among groups. The offspring weight was higher in the group treated with enrichment elements vs. control group (average 14.4 vs. 13.8 g, respectively; *p* < 0.01), a difference that was significant in females (14.1 vs. 13.4 g, respectively; *p* < 0.01) and shows a tendency in males (14.6 vs. 14.2 g, respectively; *p* = 0.07).

### 3.2. Experiment 2: Female Offspring Used as Embryo Recipients

Foster females subjected to EE showed similar reproductive parameters than females from the control group (Table 2). For both groups, pregnancy rate exceed 57% and litter size number were not significantly different (2.4 vs. 3.5, enrichment/control). The number of weaned pups per female analyzed all together or differentiated by gender was not different between experimental groups (*p* = NS). No difference was found in birth rate (i.e., number of pups from transferred embryos) between EE and the control group (*p* = 0.15).

## 4. Discussion

This study shows the effect of the enrichment of environment conditions on reproductive performance in mice, after one-year period in a Swiss Webster breeding colony and their offspring used as embryo recipient females. Previous studies in mice reported controversial findings on the effect of EE on reproductive performance. Some authors suggest that EE does not affect the reproductive outcomes of Swiss Webster and C57BL/6J mice using physical enrichment, such as plastic devices of different shapes and sizes, or cotton squares [17,18]. On the other hand, other authors reported that physical EE items and the period of exposure resulted in larger body weights of the F1 or F2 offspring generations, addressing a relevant phenotype effect [22]. In the above study, the authors found an increase in body weight of F1 males and F2 offspring, suggesting a transgenerational effect of enriched C57BL/6J adult males. Moreover, Kimura et al. [23] found differences in the pup weight at weaning in enriched mutant mice, when folded-paper nest boxes were added to the breeding cages. Our results are in agreement with the body weight increase of pups from the breeding colony, while no effect was found in other reproductive parameters. The increase in body weight of the pups may be associated with well-being of the mothers (e.g., better maternal ability) during pregnancy and lactation, as well as with well-being of the offspring promoting body growth and weight at weaning, although the effect of extra caloric source on EE group should not be discarded. This finding supports the use of this animal welfare strategy usually recommended for mice facilities, without negatively affecting general reproductive outcomes.

Regarding the effect of EE on foster mothers used as recipients for embryo transfer, it is well-known that undergone any surgery is a stressful factor that may generate consequences in the reproductive performance. It has been demonstrated that social and physical environment positively benefits post-operative recovery [24,25], and that female mice subjected to surgery need less pain relief when recovered in an enriched environment [26]. However, in our study, no differences were found in any of the parameters. Thus, at least under the conditions that this study was performed, the EE did not affect reproductive performance in foster mice.

## 5. Conclusions

In conclusion, our results show that this EE program usually recommended for improving animal welfare conditions, can be implemented in Swiss Webster mice without negatively affecting general reproductive outcomes. Environmental enrichment induced greater body weight in the offspring derived from natural mating, reinforcing the use of these kind of programs in mice breeding facilities.

## Figures and Tables

**Table 1:** Reproductive outcomes of breeder Swiss Webster mice subjected or not to enrichment conditions.

Variables	Enrichment	Control	*p*-Value
No. of females	18	18	-
No. of litters	59 ± 0.8	57 ± 0.7	NS
No. of litters/female	3.3 ± 0.3	3.2 ± 0.3	NS
Litter size (No. of pups)	7.1 ± 0.5	6.9 ± 0.4	NS
Time (d) to 1st litter	22.8 ± 0.4	24.4 ± 1.4	NS
Interlitter interval (d)	32.7 ± 1.6	36.5 ± 2.6	NS
No. of pups weaned	417	388	-
Male	220	203	-
Female	197	185	-
M: F ratio (%)	53:47	52:48	NS
Pup weight at weaning (g)	14.4 ± 0.1	13.8 ± 0.1	<0.01
Male	14.6 ± 0.2	14.2 ± 0.2	0.07
Female	14.1 ± 0.2	13.4 ± 0.2	<0.01

**Table 2:** Reproductive outcomes of Swiss Webster females used as recipients for embryo transfer.

Items	Enrichment	Control	*p*-Value
Pregnancy rate	57.1% (20/35)	68.0% (17/25)	NS
Litter size (No. of pups/female)	2.4 ± 0.4	3.5 ± 0.7	NS
No. of pups weaned	48	59	-
Male	19	26	-
Female	29	33	-
M: F ratio (%)	40:60	44:56	NS
Birth rate in total transferred females	5.7% (48/846)	10.3% (59/575)	NS
